# Rapid Screening of Psychological Well-Being of Patients with Chronic Illness: Reliability and Validity Test on WHO-5 and PHQ-9 Scales

**DOI:** 10.1155/2014/239490

**Published:** 2014-11-18

**Authors:** Shu-Fang Vivienne Wu

**Affiliations:** School of Nursing, National Taipei University of Nursing and Health Sciences, Taipei 11219, Taiwan

## Abstract

This study intended to test the reliability and validity of two simple psychological screening scales, the World Health Organization Well-being Index (WHO-5) and the 9-item Patient Health Questionnaire (PHQ-9), in patients with chronic illness in Taiwan and to understand the psychological well-being of patients with chronic illness (e.g., metabolic syndrome) in Taiwan and the incidences of psychological problems that follow. The research design of this study was a descriptive cross-sectional study. The sample comprised 310 patients with metabolic syndrome (MS), aged 20 years or more, from the outpatient clinic of a municipal hospital in Taiwan. This study used questionnaires to collect basic information, including physiological indices, WHO-5 and PHQ-9 that were used. “Hospital Anxiety and Depression scale (HADS),” and “World Health Organization Quality of Life—Short-form Version for Taiwan (WHOQOL)”. Results are as follows: (1) compared to PHQ-9, the reliability and validity of WHO-5 are better for screening the psychological well-being of patients with chronic illness. (2) The features of WHO-5 are high sensitivity, briefness, and ease-of-use. The incidence of depression in patients with metabolic syndrome was approximately 1.0–6.5%, which is significantly lower than that of western countries.

## 1. Introduction

The World Health Organization (WHO) defines chronic illness as the existence of a variety of physical health problems, which require health management for at least 1 year or even more than 10 years, such as hypertension, hyperlipidemia, and diabetes [1]. Therefore, patients with chronic illness will encounter permanent changes in health status and are more likely to be under the menace of potential death. This menace is closely related to psychological distress, such as depression. Approximately 6–34% of the patients with chronic illness developed the symptoms of depression [2]. Chronic illness is not only life-threatening to patients, but it is also a great burden to family and society. Previous studies indicated that if patients experience serious psychological distress, there may be crises to personal and family life, which increases health costs [3]. Psychological adjustment disorder will reduce long-term self-management motivation and therapeutic effect [4]. Psychosocial and cognitive factors are factors to be considered in the effectiveness of health education. Therefore, in recent years, studies on chronic illness have gradually changed their focus to the improvement and enhancement of important psychosocial factors. Metabolic syndrome belongs to one of the syndromes that consist of many chronic diseases. To prevent and delay the occurrence of complications, patients have to implement disease control and learn to adjust their living habits. Goldbacher and Matthews (2007) pointed out that metabolic syndrome is significantly correlated with psychological distress and is a typical disease covering the psychosocial aspect [5]. Previous studies found that depression is associated with certain risk factors of metabolic syndrome, such as abdominal obesity, hyperglycemia, and hyperlipidemia [6, 7].

Taking as an example the study of diabetes [8], it was found that the chief complaint of most patients (85.2%) is the diagnosis of a high level of psychological distress. Approximately 16% of the patients suffered from acute myocardial infarction, 14% suffered from congestive heart failure, and 11% suffered from diabetes [9]. In addition, after being diagnosed with disease, the psychological distress of most patients continued to exist; however, only 10% of patients with diabetes received the referral and counseling of psychological counseling. It is noteworthy that most medical and nursing personnel (61–72%) also suggested that the psychosocial well-being of their patients was poor. However, only 42% of the medical and nursing personnel were able to assess and confirm the psychological problems and needs of patients [8]. Clinically, medical and nursing personnel cannot identify the psychological problems of patients early, unless they have suitable tools or skills for assessing the psychological problems of chronic illness. The use of a scale for depression screening is a good approach. However, clinically, the symptoms of depression may be similar to those of chronic illness and may repeatedly occur, such as fatigue, weight change, and change in appetite; therefore, not all psychological scales are suitable, and it is necessary to choose them carefully. A review study reviewed articles concerning the depression of chronic patients. Among 12 studies on patients with hypertension, 9 showed that approximately 15–37% of the patients with chronic illness experienced the symptoms of depression. Among 16 studies on diabetes, 13 found that approximately 12–70% of the patients with diabetes experienced depression [10]. Well-being is an important dimension of the overall perceived quality of life and is an important outcome of chronic disease care. In people with metabolic syndrome (MS), emotional well-being may be compromised by the burden of living with their disease conditions and/or life stresses. Depression is common among persons with MS. Liu (2008) and Skilton et al. (2007) used the HADS scale as the research tool and found that 49.4% and 24.5%, respectively [11, 12], of patients with metabolic syndrome suffered from depression. Unfortunately, diagnosis of depression is often missed by health care professionals; therefore, using a short questionnaire, such as the World Health Organization Well-being Index (WHO-5), can help to monitor the emotional well-being of patients as part of the clinical routine, thus enhancing the likelihood of recognizing depression [13]. Brief instruments have been developed to screen for specific states of emotional well-being, for example, WHO-5. The WHO-5 Well-being Index is a short, self-administered questionnaire covering 5 positively worded items, which are related to positive mood (good spirits, relaxation), vitality (being active and waking up fresh and rested), and general interests (being interested in things), and has proved to be a reliable measure of emotional functioning. Administering the WHO-5 takes only 2-3 minutes and can be integrated in clinical routine. WHO-5 has the accuracy of screening questionnaires in identifying depression, as compared with the accuracy of clinical diagnosis [14].

The Patient Health Questionnaire-9 (PHQ-9) is a self-administered version of the PRIME-MD diagnostic instrument for common mental disorders and is also a reliable and valid measure of depression severity. These characteristics, as well as its brevity, make the PHQ-9 a useful clinical and research tool (Validity of a Brief Depression Severity Measure) [15]. Although both WHO-5 and PHQ-9 are perceived as brief, reliable, and valid short-form scales in western countries, whether they are suitable for use in measuring chronic illness in developing countries remains unknown.

To assess the psychological problems of patients is an enormous challenge to nursing personnel, and they must usually refer patients to professionals other than nursing personnel. Professionals should proactively provide emotional assessments to patients with chronic illness whose emotional or psychological problems cannot be effectively solved and determine whether there is a need to refer such patients to professional counseling, psychological, or mental clinics for treatment. It is necessary to improve the psychological assessment ability of professionals, as medical and nursing personnel are very busy and lack relevant skills and tools. They cannot identify patients' psychological problems early until a suitable and brief tool or skill for assessing psychological problems of diabetes is available. While considerable attention has been focused on improving the detection of depression, assessment of severity is another important guide for treatment decisions. Therefore, the objective of this study is to test the reliability and validity of two simple psychological screening scales (WHO-5 and PHQ-9) for patients with chronic illness in Taiwan and to understand the psychological well-being of patients with chronic illness in Taiwan,and the incidences of psychological problems that follow.

## 2. Methods

### 2.1. Population and Setting

Regarding research design, this study was a descriptive cross-sectional study, which used purposive sampling to choose patients who have chronic illness from the hospital clinic of a certain municipal hospital in Taipei City as the subjects and who volunteered to participate in this study. This study used a self-administered questionnaire. The inclusion criteria for the subjects are patients with at least 3 risk factors according to the definition of metabolic syndrome, as provided by Health Promotion Administration, and Ministry of Health and Welfare, Taiwan (2007) [16], including (1) abdominal obesity: male waistline ≧ 90 cm and female waistline ≧ 80 cm; (2) hypertension ≧ 130/85 mmHg; (3) fasting glucose ≧ 100 mg/dL; (4) male HDL-C < 40 mg/dL and female HDL-C < 50 mg/dL; and (5) triglyceride ≧ 150 mg/d. This is in addition to patients who were diagnosed with diabetes, were willing to participate in this study, and were aged 20 and above. The patients also had to understand and could converse in Chinese or Taiwanese. Exclusion criteria were patients with chronic illness complicated by severe complications who could not implement self-care (e.g., patients with ablepsia and patients undergoing dialysis) and patients with cognitive deficits (e.g., dementia mainly according to medical record report).

This study used statistical software G-Power to calculate the sample size [17], where *α* was set at 0.05, power was set at 0.8, and effect size was set at 0.15. The sample size should be at least 270. Moreover, because this study had to test construct validity, according to sample size calculation, at least 20 subjects should be enrolled for each item [18]. WHO-5 and PHQ-9 include 14 items in total; therefore, the sample size should be at least 280. This study was conducted between January and December 2013. Of the total of 320 patients, 310 responded to the questionnaires, which resulted in a response rate of 96%.

### 2.2. Instruction

This study used questionnaires to collect basic information, including physiological indices (BMI, biochemical tests (blood pressure, blood lipid, and blood glucose)). In terms of the psychological aspect, two questionnaires, WHO-5 (5 items) and PHQ-9 (9) items, were used for measurement. In addition, Hospital Anxiety and Depression (HADS), which has been developed for many years and has been frequently used in chronic illness, was used to test “convergent validity,” and World Health Organization Quality of Life-short-form version for Taiwan (WHOQOL) was used to test “criterion-related validity.”

### 2.3. Demographic Survey

The survey included data on gender, age, education, marital status, and other items.

*(1) The WHO-Five Well-Being Index (WHO-5).* This scale has been translated into Chinese and is available on the website of the author [19]. The WHO-Five Well-being Index (WHO-5) was developed at the Psychiatric Research Unit, Mental Health Centre North Zealand, Hillerod, Denmark. Each of the five items is rated on a 6-point Likert scale from 0 (= not present) to 5 (= constantly present). The lower the total score is, the more severe the depression, poor physical health, and psychological health are. The higher the total score, the better the physical and psychological health. An answered score of 1 or 0 on any of these items means that it may be helpful to consult with a counseling professional. A score of 13 or lower suggests further investigation into possible symptoms of depression. It is recommended to administer the major depression (ICD-10) inventory if the raw score is below 13 or if the patient has answered 0 to 1 to any of the five items. Scores are summated, with a raw score ranging from 0 to 25, and the total score is multiplied by 4 in order to obtain a percentage score, with higher scores meaning better well-being. A percentage score of 0 represents the worst possible well-being, while a score of 100 represents the best possible well-being. Evidence suggests that a score of 50 or below is indicative of low mood, though not necessarily depression. A score of 28 or below indicates likely depression and warrants further assessment (diagnostic interview) to confirm depression.

This scale was comprehensively applied to a multinational study on diabetes (DAWN), and its reliability and validity are good regarding studies on type 1 or type 2 diabetes [8, 20]. The internal consistency of the scale is Cronbach's *α* = 0.87. The test-retest reliability of this scale in Germany and Japan is Cronbach's *α* = 0.90. Therefore, this scale is suitable for use as a tool for measuring the physical and psychological health of patients with chronic illness [21].

*(2) The 9-Item Patient Health Questionnaire (PHQ-9).* The reliability and validity of this scale in patients with diabetes are good, and it can be used to rapidly screen depression [22]. This scale is available online. The content of the 9 items are simple and comprehensible, where scales from 0 (never) to 3 (almost 10 every day) are used for scoring, and the total score is 27. If the total score of a patient is ≧ 10, he/she will be advised to be referred to a clinic for major depressive disorder. The lower the total score is, the better the physical and psychological health is. The higher the total score is, the more depressive the patients are. The internal reliability of the PHQ-9 was excellent, with a Cronbach's *α* of 0.89 in the PHQ Primary Care Study. Test-retest reliability of the PHQ-9 was also excellent, and correlation between the PHQ-9 was 0.84 [15].

*(3) Hospital Anxiety and Depression (HADS).* This scale was developed by Zigmond and Snaith in 1983 for assessing emotional problems [23], such as anxiety and depression, of nonmentally ill patients in general outpatient clinics [23, 24]. A previous study indicated that most medical and nursing personnel are aware that patients may experience psychological distress; however, clinically, they seldom perform screening or assessment of problems, such as anxiety and depression, in nonmentally ill patients during the disease period [25]. HADS is a simple, reliable, and valid tool aiming for use in clinical expedited screening and has been applied in many studies around the world. It has been verified that the reliability and validity of this scale are good in community environments and primary health care. This scale has been translated into a variety of languages, such as European languages, Arabic, Chinese, and Japanese [25]. This scale consists of 14 items, including the two subscales of anxiety (7 items) and depression (7 items). In the anxiety subscale, there is 1 positive item (7) and 6 negative items (1, 3, 5, 9, 11, and 13). In the anxiety subscale, there are 5 positive items (2, 4, 6, 12, and 14), and 2 negative items (8 and 10). A 0–3 scale is used for scoring, and anxiety and depression are scored separately. If the total score is <8, the patients do not experience any anxiety or depression. If the total score is 8–10, the patients are suspected of anxiety or depression. If the total score is >11, they patients absolutely experience anxiety or depression. The correlation between the two subscales is 0.40–0.74 (average 0.56) [24]. The Cronbach's *α* of HADS-A (anxiety) is 0.68 to 0.93 (average 0.83). The Cronbach's *α* of HADS-D (depression) is 0.67–0.90 (average 0.82). The Chinese version of HADS was used to conduct this study. The Cronbach's *α* of the depression subscale was 0.82, and the Cronbach's *α* of the anxiety scale was 0.81 [26], suggesting that the stability of the Chinese version of the scale is good. The HADS scale has been translated into a Chinese version with good reliability and validity in Taiwan and has been comprehensively applied [26, 27]. This study expected that participants who reported better WHO-5 and PHQ-9 scores would also report having similar result scores when using HADS; therefore, the HADS tool was used to test convergent validity.

*(4) World Health Organization Quality of Life- (WHOQOL-BREF-) Short-Form Version for Taiwan.* WHOQOL was developed by the WHO in 1991, as based on the discussions and studies in 15 different countries or locations. The “World Health Organization Quality of Life” (WHOQOL-100) was completed in 1995. This questionnaire can be divided into general dimensions and six major categories, including physiology; psychology; and level of independence, social relationship, environment, and spiritual, religious, and personal beliefs. The content includes a total of 24 dimensions. The items can currently measure the objective feelings or subjective self-assessment of respondents [28]. In order to use this scale in clinical trial or epidemiology investigations, WHOQOL-100 was simplified as a short-form version that consists of 26 items, called WHOQOL-BREF. In other words, one item was chosen from each of the 24 dimensions and 2 items were chosen from the general dimension. In addition, the items were classified into 4 categories: physiological health, psychological health, social relationships, and environment [28]. The 28-item WHOQOL-BREF was used in this study. The Cronbach's *α* of the total scale was 0.91, and the test-retest reliability was 0.75–0.91. The Cronbach's *α* of various scales is 0.70–0.77 [29, 30]. The study on patients with metabolic syndrome found that the internal consistency Cronbach's *α* of this scale was 0.89 [31]. A 5-point scale was applied to this scale, where the higher the score, the better the quality of life. This study expected that participants' depression would predict poor quality of life of patients with chronic diseases; therefore, WHOQOL was chosen to test the criterion validity of WHO-5 and PHQ-9.

### 2.4. Statistical Analysis

Data were double-entered for verification using SPSS 20.0 statistical software, and regression analysis was performed to explore how well WHO-5 and PHQ-9 could explain the WHOQOL for criterion validity (predictive validity). Internal consistency was assessed by calculating Cronbach's alpha for WHO-5 and PHQ-9. The content validity index (CVI) was examined by undertaking content validity. Pearson correlation coefficients between WHO-5, PHQ-9, and HADS were selected in order to examine the convergent validity of these two scales.

## 3. Ethical Considerations

The study was approved by the Institutional Review Board (IRB) for the protection of the participants' rights. Participants were given detailed information regarding the study procedures, and written consent was obtained. To ensure the subjects' rights and ethical considerations, descriptions comprehensible to the subjects were used to explain the research objectives, methods, expected benefits, and potential risks. In addition, the subjects signed an informed consent form on their own after giving their consent. All participants in this study were voluntary, and participants were allowed to withdraw from the project during the period of the study without penalty. Personal information of potential participants was keyed into a secure database maintained by the research team.

## 4. Results

### 4.1. Demographic Data

This study enrolled a total of 310 subjects, with 159 male subjects (51.3%). The average age of the subjects was 64.16 (SD = 11.11), and the average BMI was 26.00 (SD = 4.56). The BMI of 107 subjects was <24, suggesting that the weights of 107 subjects (35.4%) were normal. The BMI of 95 subjects was 24.1–26.9, suggesting that 95 subjects (31.5%) were overweight. The BMI of 100 subjects was >27, suggesting that 100 subjects (33.1%) were obese. The average waistline was 90.11 cm (SD = 10.87), and the average HbA1c was 7.67 mg/dL (SD = 1.63). In terms of education levels, 103 subjects (33.3%) were elementary school. The subjects suffered from diabetes for an average of 10.86 years (SD = 9.26). The subjects suffered from hypertension for an average of 5.83 years (SD = 7.26). The subjects suffered from hyperlipidemia for an average of 2.83 years (SD = 5.05). The remaining subjects are as shown in Table 1.

### 4.2. Reliability and Validity of WHO-5

*(1) Content Validity.* There were 5 clinical experts performing the CVI test regarding the expert validity of WHO-5, and the scale was assessed according to content appropriateness. The 5-point Likert Scale (1 denotes strongly disagree, while 5 denotes strongly agree) was used for scoring. The expert validity CVI of the scale was an average of 0.76–0.8. The average total score was 0.77, suggesting that the content validity of the scale was good (as shown in Table 2). The expert panel did not modify the content or the working scale regarding the scale of WHO-5.

*(2) Construct Validity.* Convergent validity was used to test construct validity. Convergent validity can be established if two similar constructs correspond with one another and can be estimated using correlation coefficients. A successful evaluation of convergent validity shows that the tested concept is highly correlated with other tests designed to measure theoretically similar concepts. This study used the “Hospital Anxiety and Depression Scale (HADS-Depression),” which has mature development and has been frequently used in chronic illness to test convergent validity. The lower the score of WHO-5 is, the more the patients suffer from severe depression. In this study there was negative correlation between them (*r* = −0.57, *P* < 0.01), suggesting that the convergent validity and construct validity were high.

*(3) Criterion-Related Validity.* Criterion-related validity is a measure of how well one variable, or set of variables, predicts an outcome based on information from other variables. The study on patients with metabolic syndrome found that the quality of life for patients with metabolic syndrome is poor, due to the influences of the burdens of depression, obesity, and disease [32]. Therefore, depression was significantly correlated with quality of life. A previous study indicated that, compared to a low risk group, the quality of life for patients with type 2 diabetes, and thus a high risk group, was lower, and their depression score was higher [33]. Therefore, WHO-5 and WHOQOL were used to test the relationship at this stage. WHO-5 was a significant predictor of quality of life using regression analysis (*r* = 0.49, *P* < 0.001) and accounted for 24.2% of the variance in the total WHOQOL scores. In other words, it is predictable that the quality of life of patients with good physical and psychological health is better. This result suggests that WHO-5 measures depression and WHO-5 is a predictor of quality of life; therefore, the criterion-related validity was good.

*(4) Consistency Reliability.* Regarding reliability, according to analysis of the 310 enrolled subjects, the Cronbach's alpha of this scale was 0.89, suggesting that the internal consistency of WHO-5 was good.

### 4.3. Reliability and Validity of PHQ-9

*(1) Content Validity.* There were 5 clinical experts performing the CVI testing of PHQ-9. The total average of CVI of this scale was 0.74, suggesting that the content validity was good. The contents that the experts advised to be revised were as follows. Item 6 was originally “I dislike myself, and feel that I perform poorly and fail to meet the expectations of my family,” and it was advised to be revised as “Feeling bad about myself - or that you are a failure or have let yourself or your family down.” Item 8 was originally “Other people suggest that you will become restless because your actions are slow or you speak slowly,” and it was advised to be revised as “Moving or speaking so slowly that other people could have noticed? Or the opposite: being so fidgety or restless that you have been moving around a lot more than usual.” Item 9 was originally “I feel that I would better kill or hurt myself,” and it was advised to be revised as “Thoughts that I would be better off dead or of hurting yourself in some way” (as shown in Table 3). There were 3 items (6, 8, and 9), which were slightly revised to complete the content validity of PHQ-9.

*(2) Construct Validity.* Correlation coefficients of PHQ-9 and HADS-D (depression) were used to reflect convergent validity (*r* = 0.52, *P* < 0.01). The higher the score of PHQ-9 and HADS-D is, the more patients suffer from severe depression, and there was a significantly positive correlation between them. Therefore, the results of depression of these two scales were consistent, suggesting that the construct validity was good.

*(3) Criterion-Related Validity.* PHQ-9 was a significant predictor of quality of life, which used regression analysis (*r* = 0.40, *P* < 0.001), and accounted for 16.0% of the variance in the total WHOQOL scores. Significant results were found between PHQ-9 and WHOQOL. This result suggests that PHQ-9 measures depression and is a good predictor of quality of life. Therefore, the criterion-related validity of PHQ-9 was good.

*(4) Consistency Reliability.* The Cronbach's alpha of the scale was 0.80, suggesting that the internal consistency of the scale was good.

### 4.4. Correlation of Physical and Psychological Health of Patients with Chronic Illness and Incidences of Psychological Problems

*(1) Correlation Analysis on WHO-5, PHQ-9, HADS-A, and HADS-D.* This study found that WHO-5 was not correlated with basic information; however, it was positively correlated with WHOQOL  (*r* = 0.49, *P* < 0.01), suggesting that the better the physical and psychological health is, the better the quality of life is. PHQ-9 was natively correlated with WHOQOL  (*r* = −0.40, *P* < 0.01), suggesting that the poorer the physical and psychological health is, the poorer the quality of life is (Table 4).

*(2) Incidences of Psychological Problems in Patients with Chronic Illness.* This study used WHO-5 to perform screening and found that 5.5% of the subjects experienced emotional distress, while 1.0% confirmed suffering from depression, and 6.5% of the subjects were screened with depressive emotions. This study used PHQ-9 to perform screening and found that 4.8% of the subjects suffered from depression. This study used HADS-A, which has been frequently used, to perform screening and found that 1.9% of the subjects were suspected of anxiety, 0.6% were identified as patients with anxiety, and 2.5% had a tendency to anxiety. This study used HADS-D to perform screening and found that 3.5% of the subjects were suspected of depression, 1.0% was identified as patients with depression, and 4.5% had a tendency to depression. The above findings showed that the incidences of depression and anxiety, as measured by WHO-5 and HADS-D, were extremely consistent (1%). The screening results showed a consistent value (Table 5); therefore, compared to PHQ-9, WHO-5 seemed to be more sensitive in the screening of depression.

## 5. Discussion

Convergent validity is generated from correlations between two different tools measuring the same trait [34]. This study found that WHO-5 and PHQ-9 were correlated with the score of the clinically practical HADS scale, suggesting that they were indeed used to measure the same concept—depression. Therefore, it could be explained that the construct validity of WHO-5 and PHQ-9 was good.

Predictive validity can be tested by examining the ability to predict the current value of one measure based on the value obtained on the measure of another concept. Psychometric testing established the psychometric properties of WHO-5 and PHQ-9. The assumption underlying this study indicates that depression is a useful predictor for quality of life. Results show that WHO-5 and PHQ-9 are significant predictors of quality of life. Good psychological health could be used to predict that the quality of life of patients was good. These findings were similar, which show that depression accounts for an average WHO-5 (24.2%) and PHQ-9 (16%) of the variance in quality of life, which demonstrates that depression is a significant predictor of quality of life. Therefore, the WHO-5 and PHQ-9 instruments performed as predicted for criterion validity testing.

The reliability of WHO-5 and PHQ-9 was high, with values of 0.89 and 0.80, respectively, which are sufficient for assessment at the individual level [35]. These values are similar to western countries [21]. (Cronbach's alpha 87~.90).

In terms of incidences of depression, the incidences of depression in WHO-5 and HASD-D were both 1%, and the screening results were consistent. Therefore, this study found that, compared to PHQ-9, WHO-5 was more sensitive in the screening of patients with depression. The various psychological screening scales used in this study found that the incidences of a tendency of depression in patients with metabolic syndrome were 1.0–6.5%, which were the same as the value (6–34%) found by the study on patients with chronic illness by Leung (1998) [2]. However, the results were different from those of relevant studies of metabolic syndrome in western countries. Skilton et al. (2007) used HADS as the research tool and found that 24.5% of the patients were plagued by depression [12]. Gary et al. (2000) used the CES-D scale as a research tool and found that 23.84% of the patients were plagued by depression [36]. Moreover, a study using BDI as the research tool found that 10.6% of the patients were plagued by depression [37], and the difference might be caused by the differences in subject population, characteristics, and enrollment site. The study site of this study was an outpatient clinic, and the subjects were able to care for themselves in daily life; thus, their disease severity might be milder. Therefore, their psychological barriers were less significant, leading to a lower incidence rate of depression in this study than that of other studies in western countries. Moreover, the rate of severe and mild depression seems rather low in patients with a metabolic syndrome in this study. This might be caused by the following reasons. (1) Patients with MS were able to care for themselves and accept their illness and do not experience unhappiness or discontent. Such a mental state might explain the relatively low percentage of depression occurrence. (2) Hispanic and Native American patients with chronic disease are more susceptible to depression, whereas Asians are less susceptible [38]. (3) Most patients are of middle or old age with minimum education. Such a subpopulation has low awareness of mental health and does not acknowledge the important connection between mental and physical health. Che et al. (2006) pointed out that Chinese people tend to suppress their outward emotional responses, resulting more commonly in physically oriented expressions of emotion. The depression and stress are mostly manifested in physical disorders [39]. Similarly, according to a study on psychological problems of patients with diabetes by Wu et al. (2012) [40], approximately 10.6% of patients with diabetes suffered from depression, which was significantly lower than that in other foreign studies. The reason might be that Chinese people are more conservative and tend not to express their depressive emotions.

The psychological health of patients with metabolic syndrome may easily lead to functional deficits and increased medical costs; therefore, future studies are advised to perform further investigation. The specific suggestions are as follows: (1) to strengthen medical and nursing personnel's preliminary assessment and intervention of psychosocial problems of patients (e.g., use of brief depression and anxiety scales with reliability and validity for screening); (2) to implement referral of patients with severe depression to professional psychological facilities; (3) to increase group health education using psychological cognition as the framework; (4) to train professional caregivers and strengthen psychological counseling training courses; and (5) to develop studies regarding the psychology of chronic illness (i.e., future studies may use experimental design, effectiveness of psychological intervention measures, and qualitative research design to develop studies regarding the psychology of chronic illness).

## 6. Conclusions

Results of this study support the validity and reliability of WHO-5 and PHQ-9 in providing a measure of depression for persons with chronic disease in Taiwan, especially WHO-5, which is a 5-item, sensitive, brief, and effective tool for screening depression caused by chronic illness. It is relatively short and is easy to administer to the Chinese population with chronic disease, as it requires only 10 minutes for completion.

## 7. Research Limitations

Since use of nonprobability sampling from one clinic may be thought to limit the generality of the findings, nevertheless, the results are of major importance and significance to the local populations of a developing country (Taiwan).

## Figures and Tables

**Table 1:** Patients' basic attributes (*n* = 310).

Variables	Max/min	Mean	Std. deviation	*n*	%
Age	90,31	64.16	11.11		
BMI	52,17	26.00	4.56		
Waistline	129.0,62.5	90.11	10.87		
HbA1c	15.7,5.1	7.67	1.63		
Number of years of disease experience					
Diabetes	45,0	10.86	9.26		
Hypertension	40,0	5.83	7.26		
Hyperlipidaemia	30,0	2.83	5.05		
BMI classification					
BMI <24 (normal)				107	35.4
BMI 24.1–26.9 (overweight)				95	31.5
BMI >27 (obese)				100	33.1
Gender					
Female				151	48.7
Male				159	51.3
Education level					
Under elementary school				103	33.3
Junior high school				54	17.4
Senior high school and vocational senior high school				73	23.5
Junior college				27	8.7
University and above				53	17.1
Categories of complications					
No				37	11.9
Yes				273	88.1

**Table 2:** Expert validity of World Health Organization's scale of 5-item physical and psychological health indices.

Results	Content appropriateness	CVI average score
(1) I have felt cheerful and in good spirits	20	0.80
(2) I have felt calm and relaxed	19	0.76
(3) I have felt active and vigorous	20	0.80
(4) I wake up feeling fresh and rested	19	0.76
(5) My daily life is filled with things that interest me	19	0.76

Total average		0.77

**Table 3:** Expert validity of scale on patients' health status (PHQ-9).

Results	Content appropriateness	CVI average score
(1) Little interest or pleasure in doing things	19	0.76
(2) Feeling down, depressed, or hopeless	19	0.76
(3) Trouble falling/staying asleep, sleeping too much.	20	0.80
(4) Feeling tired or having little energy	20	0.80
(5) Poor appetite or overeating	19	0.76
(6) Feeling bad about yourself—or that you are a failure or have let yourself or your family down	18	0.72
(7) Trouble concentrating on things, such as reading the newspaper or watching television	20	0.80
(8) Moving or speaking so slowly that other people could have noticed? Or the opposite—being so fidgety or restless that you have been moving around a lot more than usual	15	0.60
(9) Thoughts that you would be better off dead or of hurting yourself in some way	17	0.68

Total average		0.74

**Table 4:** Results of correlation analysis on WHO-5, PHQ-9, HADS-A, and HADS-D (*n* = 310).

Variables	WHO-5	PHQ-9
Age	0.07	−0.06
BMI	−0.04	−0.01
Waist	−0.05	−0.03
HbA1C	−0.08	0.06
DM-years	−0.01	0.02
HT-years	0.03	0.03
HC-years	−0.03	0.07
WHOQOL	0.49^**^	−0.40^**^
WHO-5	1	−0.60^**^
PHQ-9	−0.60^**^	1
HADS-A	−0.42^**^	0.61^**^
HADS-D	−0.57^**^	0.52^**^

^**^ *P* < 0.01.

**Table 5:** Incidences of brief psychological screening scale (*n* = 310).

Scores of indices	Number of subjects	Screening rate (%)
World Health Organization's scale of 5-item physical and psychological health indices (WHO-5)		
Depression (score 1–28)	3	1.0
Emotional distress (score 29–50)	17	5.5
Normal (score 51–100)	290	93.5
Patient health questionnaire (PHQ-9)		
Normal (score ≦10)	295	95.2
Depression (score >10)	15	4.8
Hospital anxiety and depression scale-anxiety (HADS-A)		
Normal (score 0~7)	302	97.4
Suspected anxiety (score 8~10)	6	1.9
Identified anxiety (score ≧11)	2	0.6
Hospital anxiety and depression scale-depression (HADS-D)		
Normal (score 0~7)	296	95.5
Suspected depression (score 8~10)	11	3.5
Identified depression (score ≧11)	3	1.0
